# Acceptability and Preliminary Efficacy of a Web- and Telephone-Based Personalised Exercise Intervention for Individuals with Metastatic Prostate Cancer: The *ExerciseGuide* Pilot Randomised Controlled Trial

**DOI:** 10.3390/cancers13235925

**Published:** 2021-11-25

**Authors:** Holly E. L. Evans, Daniel A. Galvão, Cynthia C. Forbes, Danielle Girard, Corneel Vandelanotte, Robert U. Newton, Andrew D. Vincent, Gary Wittert, Ganessan Kichenadasse, Suzanne Chambers, Nicholas Brook, Camille E. Short

**Affiliations:** 1 Freemasons Centre for Male Health & Wellbeing, School of Medicine, University of Adelaide, Adelaide 5001, Australia; andrew.vincent@adelaide.edu.au (A.D.V.); gary.wittert@adelaide.edu.au (G.W.); camille.short@unimelb.edu.au (C.E.S.); 2 Exercise Medicine Research Institute, Edith Cowan University, Joondalup 6027, Australia; d.galvao@ecu.edu.au (D.A.G.); r.newton@ecu.edu.au (R.U.N.); Suzanne.Chambers@acu.edu.au (S.C.); 3 Wolfson Palliative Care Research Centre, Institute of Clinical and Applied Health Research, Hull York Medical School, University of Hull, Hull HU6 7RX, UK; cindy.forbes@hyms.ac.uk; 4 Alliance for Research in Exercise, Nutrition and Activity, Allied Health and Human Performance, University of South Australia, Adelaide 5001, Australia; danielle.girard@unisa.edu.au; 5 Physical Activity Research Group, Appleton Institute, Central Queensland University, North Rockhampton 4702, Australia; c.vandelanotte@cqu.edu.au; 6 School of Human Movement and Nutrition Sciences, The University of Queensland, Brisbane 4067, Australia; 7 College of Medicine and Public Health, Flinders Centre for Innovation in Cancer, Flinders University, Bedford Park 5042, Australia; Ganessan.Kichenadasse@flinders.edu.au; 8 Faculty of Health Sciences, Australian Catholic University, Brisbane 4041, Australia; 9 Department of Surgery, School of Medicine, University of Adelaide, Adelaide 5001, Australia; nicholasbrook@gmail.com; 10 Melbourne Centre for Behaviour Change, Melbourne School of Psychological Sciences, The University of Melbourne, Melbourne 3010, Australia; 11 Melbourne School of Health Sciences, The University of Melbourne, Melbourne 3010, Australia

**Keywords:** exercise, metastatic prostate cancer, behavioural change, eHealth, computer-tailoring, usability, acceptability, rct

## Abstract

**Simple Summary:**

Previous research supports the participation in supervised exercise among individuals with metastatic prostate cancer to help lessen the physical and psychological disease burden. However, many individuals experience considerable barriers to attending face-to-face exercise services. To overcome some of these limitations, digital interventions that can be delivered remotely have been proposed. Our pilot study investigated the acceptability, safety and preliminary efficacy of an 8-week computer-tailored web-based exercise intervention. We demonstrated that a web-based exercise program with telehealth support was acceptable and could be implemented safely. Participants in the intervention group increased their participation in moderate to vigorous physical activity compared to the control group. This study provides insight into the prospect of web-based exercise prescription for individuals with metastatic prostate cancer as an alternative for individuals who cannot access supervised exercise interventions.

**Abstract:**

Preliminary research has shown the effectiveness of supervised exercise-based interventions in alleviating sequela resulting from metastatic prostate cancer. However, many individuals encounter barriers that limit the uptake of face-to-face exercise. Technology-enabled interventions offer a distance-based alternative. This pilot study aimed to explore the acceptability, safety and preliminary efficacy of a web-based exercise intervention (*ExerciseGuide*) in individuals with metastatic prostate cancer. Forty participants (70.2 ± 8.5 years) with metastatic prostate cancer were randomised into the 8-week intervention (*N* = 20) or a wait-list control (*N* = 20). The intervention arm had access to a computer-tailored website, personalised exercise prescription and remote supervision. *ExerciseGuide* was deemed acceptable with a score ≥20 on the client satisfaction questionnaire; however, the usability score was just below the pre-specified score of ≥68 on the software usability scale. There were no serious adverse events reported. Moderate-to-vigorous physical activity levels between baseline and follow-ups were significantly higher (10.0 min per day; 95% CI = (1.3–18.6); *p* = 0.01) in the intervention group compared to wait-list control. There were also greater improvements in step count (1332; 95% CI = (159–2505); *p* = 0.02) and identified motivation (0.4, 95% CI = (0.0, 0.7); *p* = 0.04). Our findings provide preliminary evidence that *ExerciseGuide* is acceptable, safe and efficacious among individuals with metastatic prostate cancer.

## 1. Introduction

Prostate cancer is the second most common malignancy in Australia, with one in six individuals diagnosed by the age of 85 [1]. Unlike individuals with localised prostate cancer who have a 95% five-year survival, individuals whose cancer has metastasised to secondary sites (bone, viscera and nodes) have an approximately 30% five-year survival despite treatment [1].

Individuals with metastatic prostate cancer face substantial physical and psychological deterioration due to toxicities relating to life-prolonging therapies such as treatment with abiraterone, enzalutamide, docetaxel, cabazitaxel, radium-223 and sipuleucel-T [2]. Furthermore, individuals deal with symptoms from the disease, such as pain and disability relating to metastasis, which are typically found in bone, liver and thorax [3]. These negative changes create a vicious cycle of physical inactivity, which then accelerates their physical decline [4]. It has been established that individuals with metastatic prostate cancer have a desire to maintain sufficient physical and emotional functioning to maintain their daily responsibilities (i.e., physical housework, hobbies and spending time with loved ones); it is essential that their physical function and cardiorespiratory fitness is retained for as long as possible in order to maximise quality of life [5]. 

Strong evidence supports tailored exercise prescription in individuals with metastatic prostate cancer within supervised research settings [4,6,7]. Researchers have stated improved physical function, body strength, submaximal aerobic exercise capacity, ambulation, lean mass and emotional well-being [8,9,10]. Although it is still unclear what effect a tailored exercise program has on long term survival in this population, observational studies have reported that increased physical activity is associated with a lower risk of cancer-specific mortality in individuals with localised prostate cancer [11,12]. 

Despite the significant positive benefits observed, the gold-standard but resource-intensive model of tailored supervised exercise seen in research settings and exercise clinics is unlikely to be accessible for all individuals. For example, Sheill et al. reported that individuals with metastatic prostate cancer can face considerable financial, access and time limitations, as well as treatment-related side effects that can impede supervised exercise uptake and adherence in community settings [13]. Moreover, individuals within the Sheill et al. study noted that lack of appropriate exercise-based facilities in rural areas was also a notable barrier [13]. 

To address some of these barriers, technological advances, such as computer-tailored websites (where content is personalised using algorithms), present alternative exercise prescription and education opportunities. In other oncology populations, web-based interventions have shown promising behaviour changes in diet and physical activity [14,15,16]. The advantage of web-based technology lies in the added scalability, reach, cost-effectiveness and accessibility [16]. No studies have focused on utilising detailed algorithms to tailor both aerobic and resistance training prescriptions to individuals with metastatic prostate cancer, but the opportunities to provide adaptive distance-based individualised exercise prescription is especially valuable. Compared to individuals with localised disease, individuals with metastatic prostate cancer often have bone lesions and other considerations for exercise prescription, which increase the need for tailored programming to reduce risks. The lab-based evidence of computer-tailored exercise prescription shows encouraging acceptability, usability and safety levels in individuals with metastatic prostate cancer; however, feasibility, safety and efficacy in a real-world setting is currently unknown [17]. 

To be effective in the real-world, some level of human contact is likely needed. Previous research suggests that having someone to be accountable to is important for adherence to web-based programs, even when computer-tailoring is employed to personalise context [18]. A recent review of web-based programs supports this, with web-based programs that incorporate human support found to have greater efficacy and adherence than those that are standalone [19]. 

We undertook a pilot randomised controlled trial to assess the acceptability, safety and preliminary efficacy of a computer-tailored web-based intervention (*ExerciseGuide*) in combination with brief tele-support for enhancing exercise and health among individuals with metastatic prostate cancer. 

## 2. Materials and Methods

### 2.1. Trial Design

A randomised controlled pilot trial comprising two study arms, (1) an 8-week exercise intervention arm (web-based tool and telehealth support) and (2) a wait-list control arm. The study protocol, including criteria for progression to a larger definitive trial, was published a priori [17]. Assessments are completed at baseline and in week nine. Ethics approval was granted by the University of Adelaide Research Ethics Committee and two South Australian Health Human Research Ethics Committees (Southern Adelaide Local Health Network and Central Adelaide Local Health Network). The study was registered on the Australian New Zealand Clinical Trials Registry (ACTRN12618001979246) and reporting and conduct adhered to the Consolidating Standards of Reporting Clinical Trials (CONSORT) guidelines [20]. Due to the nature of the study, it was not feasible to blind participants. All participants provided written informed consent to participate before entering the study.

The *ExerciseGuide* trial was conducted Australia-wide, with protocols described in depth previously [21]. In short, mixed methods (questionnaires, accelerometry, qualitative interviews and physical function testing) were used for acceptability, safety and efficacy evaluation, with the success of the intervention interpreted based on the following criteria:

1. Acceptability:
  a. The acceptability of the intervention was satisfactory (an average score of ≥20 on the client satisfaction questionnaire) [22].
  b. The website usability was satisfactory (an average score of ≥68 on the software usability scale) [23].
2. Safety:
  a. No grade 3+/life threatening, or severe adverse events resulted from participating in the intervention.
3. Efficacy:
  a. Evidence of clinically meaningful participation in either aerobic and or resistance exercise in the intervention group relative to the wait-list control, defined as a between-group difference of at least 30 min of aerobic activity and or one resistance training session per week.
4. Feasibility of conducting a larger-scale trial:
  a. The recruitment goal of 66 participants was reached.
  b. Behaviour change data was collected for ≥75% of participants.
  c. Physical functioning data was collected for ≥75% of participants that (a) reside within 30 km from a study testing site and (b) are invited to complete testing.

Additional measures of acceptability, safety and efficacy were also included and are described in detail below.

### 2.2. Participants

One hundred and forty-one male patients with metastatic prostate cancer expressed interest in the study from February 2020 to January 2021 and forty-one were deemed eligible to participate. Participants met the inclusion criteria if they had a confirmed diagnosis of metastatic prostate cancer and medical clearance to participate in the study from their physician (i.e., General Practitioner, Medical Oncologist), which also detailed all metastasis location/s and severity. Participants were required to be proficient in English, have access to the internet and be free from contraindications to performing moderate intensity exercise (i.e., no recent serious cardiovascular events within 12 months, unstable bone metastases, spinal compressions or acute illnesses) [24].

Participants were excluded if they were deemed as currently sufficiently active, which was defined as already engaging in two sessions of resistance training and 60 min of structured moderate–vigorous aerobic exercise per week or had current moderate to severe bone pain (Common Terminology Criteria for Adverse Events V.5.0 grading criteria).

### 2.3. Recruitment

Participants were recruited via social media advertisements (i.e., Facebook), Prostate cancer and men’s health research volunteer registries (Prostate Cancer Foundation of Australia Pathfinders registry and The Freemasons Centre for Male Health and Wellbeing registry), the South Australian Prostate Cancer Registry prostate cancer nurses, urologists and medical oncologists. All potential participants were directed to the study webpage (www.exerciseguide.org.au; accessed 23 November 2021). Detailed study information, an automated screening tool and researchers’ contact details were found on the website. Interested participants were encouraged to complete the automated web-based screening survey, and if assessed as potentially eligible, were asked to provide contact details. Researchers then verified eligibility via a phone interview, and an information pack was emailed or posted. Once written consent and medical clearance was returned, baseline assessments were undertaken.

### 2.4. Randomisation

Participants were stratified by age (≤65 years, >65 years) and physical function (physical functioning domain of the European Organisation for Research and Treatment of Cancer (EORTC) quality of life questionnaire ≤80, >80). After the baseline assessments were completed, participants were randomised into either the *ExerciseGuide* intervention group or the wait-list control group using block randomization (block sizes of 2 and 4).

### 2.5. Intervention

#### 2.5.1. *ExerciseGuide* Intervention

**Figure 1:**
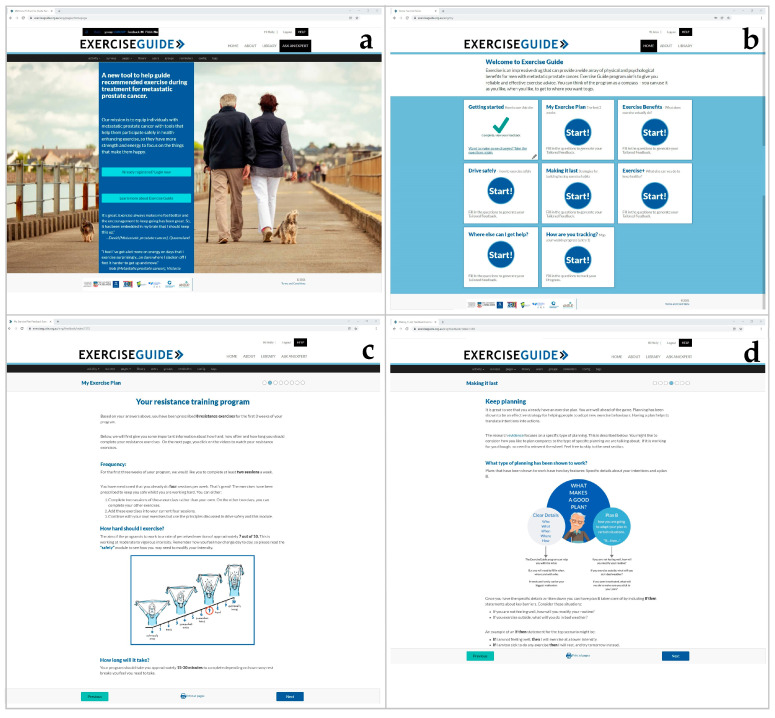
Screenshots of the *ExerciseGuide* intervention: (**a**) the *ExerciseGuide* landing page, (**b**) the *ExerciseGuide* homepage, (**c**) the My Exercise Plan 1 module (**d**) the Making it Last (behaviour change) module.

A detailed description of the *ExerciseGuide* intervention has been published previously [21]. Briefly, participants received access to a computer-tailored website (www.exerciseguide.org.au; accessed 23 November 2021), which provided individualised multi-modal exercise prescription via text and videos and behavioural change information for eight weeks in the form of nine computer-tailored modules (see Figure 1). All modules (with exception of the weekly tracking modules) were accessible all at once (free-choice design) in order to promote autonomy and allow users to self-tailor usage according to their interests. The weekly tracking module opened after the first seven days and once completed the next tracking module opened seven days later. The modules were developed from previous exercise oncology [7,8,25] and behavioural science studies [5,8,26], and were guided by behavioural change theories [27,28,29,30,31,32,33]. The modules included:

1. Getting started (how to use the website and basics of the intervention);
2. My exercise plan 1 (tailored exercise programming for week 1–3);
3. My exercise plan 2 (progression/regression tailored exercise programming for week 4–8);
4. Exercise benefits (health benefits specific to metastatic prostate cancer);
5. Exercise safely (considerations necessary to remain safe whilst exercising);
6. Making it last (behavioural change information, including building confidence and habits);
7. Exercise + (information on nutrition, distress, sedentary behaviours, sleep, etc.);
8. Where else can I get help? (Facilitate access to additional support);
9. How are you tracking? (Facilitate self-monitoring of exercise behaviours and exercise outcomes).

The *ExerciseGuide* website also contained a library, with short prostate cancer, exercise or behaviour change articles, written in layman’s language and an “Ask the EP” (Exercise Physiologist) feature of the website, where participants have the option of submitting questions to the exercise physiologist.

Participants were provided with an individualised aerobic, resistance-based and flexibility exercise prescription based on data they entered into the website in week one and week three as described in depth in the study protocol [21]. Participants were also able to regress or progress their prescription at any time by changing the answers to the computer-tailoring questions within the module. The prescriptions were based upon exercise guidelines for cancer patients [25], and approaches used in previous studies for managing bone lesions [8,9,34]. In short, the aerobic training prescription ranged from 2–3 sessions per week for 16–40 min (including rest breaks when prescribed). Regressions and progressions were made by manipulating duration and mode. The resistance training prescription ranged from 2–3 days per week and included 2–3 sets of 8–12 repetitions with a focus of strength and hypertrophy. The program aimed to slowly increase session volume until week six, where volume was reduced, and an emphasis was placed on increasing load to maintain intensity. This change was prescribed to reduce the likelihood of participants increasing repetition range past 12 repetitions as a method of progression, which would likely extend session duration without significant improvements in strength. The OMNI resistance and aerobic exercise scale of rate perceived exertion (RPE) was used to prescribe intensity, which ranged from 6 to 7 out of 10. Participants were instructed to increase or decrease the speed (aerobic training) or load (resistance training) of the exercises by a subjective 5–10% if RPE scores did not match the prescribed OMNI scale [14]. Four resistance exercise bands with variable levels of tension and a door anchor were supplied. Participants with access to home-based or gym resistance training equipment were encouraged to replicate the exercises if the equipment was suitable. A paper-based exercise diary was also provided to allow self-reporting and tracking of resistance exercises (exercises, sets, repetitions, session RPE, duration, bone pain visual analogue score and general pain visual analogue scale), aerobic exercises (type, duration, session rate of perceived exertion, bone pain score and general pain scale) and stretching exercises performed.

Additionally, participants received two telehealth consultations (week 1 and 4) using a videoconferencing platform such as Zoom [35], Skype [36] or phone calls. Consultation mode was determined by participant preference. Within these sessions, behaviour change considerations that included barriers and confidence were discussed, and participant’s exercise prescription was reviewed and modified based on clinical judgement if required). The consultations were conducted by HELE, who is an accredited exercise physiologist with seven years of experience. In weeks 2–3 and 5–8, participants were contacted by short message service (SMS) or email based on their preference to monitor compliance, aid adherence and provide support when needed.

#### 2.5.2. Wait-List Control Intervention

Participants in the wait-list control group received no specific instructions regarding physical activity or access to equipment during the eight-week intervention. After follow-up outcome measures were assessed, the wait-list control group were offered the full *ExerciseGuide* intervention and were also supplied resistance bands

### 2.6. Outcome Measure

Unless otherwise specified, outcome measures were assessed at baseline (0 weeks) and at follow-up (week 9).

#### 2.6.1. Acceptability

For the purposes of this trial, acceptability was viewed as a multi-faceted construct related to user thoughts and feelings about the intervention. Several measures of acceptability were included, with priority given to facets that were thought to potentially influence efficacy or future uptake [30,37]. All acceptability measures were assessed in intervention participants only and only at follow-up, with the exception of star ratings and comments [37] for modules, which were collected in real-time and were optional. For all measures, higher scores indicate higher acceptability.

The client satisfaction questionnaire-8 (CSQ-8) was used to evaluate overall intervention satisfaction. Eight items were measured on a 4-point scale [22]. The cut-point of 20 was chosen to designate satisfaction as it represents an average score of 2.5 on each item. The perceived usability of the intervention website was evaluated using the System Usability Scale. A score above 68 is considered “above average” and was chosen as the cut-point for success [23].

The perceived environmental supportiveness scale (15 items) was used to assess perceptions of need support provided by *ExerciseGuide* [38]. The measure provides an overall score and autonomy, structure and involvement subscale scores [38].

The extent to which the intervention was considered personally relevant was assessed using three items (“the program was very relevant to me”, “the program was very applicable” and “the program seems like it was written for someone like me in mind”) on a 7-point scale ranging from strongly disagree to strongly agree.

Finally, three open-ended survey questions asking for feedback on the positives of the program, constructive feedback and suggestions for improvement were used to understand users’ perception [39].

#### 2.6.2. Website Usage

Website usage was measured using the Google Analytics web traffic analysis platform and built-in website tracking software [40].

#### 2.6.3. Safety

Two methods assessed the safety of the exercise program. Participants were instructed to report any adverse events in the study period to the study project coordinator (HELE). Information collected included date event occurred, date reported and grade based on the Common Terminology Criteria for Adverse Events V.5.0 grading criteria. An adverse events item (“Do you feel that you have experienced any health issues as a result of participating in this research study”) was also included within the follow-up questionnaire based on the same criteria.

#### 2.6.4. Efficacy

##### Behaviour Change–Physical Activity and Exercise

The ActiGraph GT3X activity monitor (Actigraph, Pensacola, FL, USA) was used to objectively measure minutes of moderate to vigorous physical activity (MVPA) as well as light physical activity and sedentary time minutes at baseline and follow-up. Participants were mailed and asked to wear the devices on the right hip for seven days and only remove during water-based activities and sleep [40]. The validity and reliability of the ActiGraph GT3X has been established [41]. Triaxial data was recorded in 1-s epochs for at least 600 min of wear time per day on at least five days within a seven-day period, and wear-time was validated using Choi et al. [42]. The Godin leisure-time exercise questionnaire assessed self-reported physical activity (average frequency and duration of mild, moderate and vigorous aerobic exercise and total resistance training) at baseline and follow-up [43,44].

Adherence to the prescribed exercise program was assessed by auditing participant’s exercise diaries, which included self-reported information on the frequency, intensity, time and type of exercise completed. This was then cross-referenced with their individualised prescription. Perceptions of adherence were also assessed using two items (“I have been doing all of the aerobic [cardiovascular] exercises I was asked to by *ExerciseGuide*” and “I have been doing all of resistance-based [strength] exercises I was asked to by *ExerciseGuide*”) with an 11-point numeric rating scale (0 = strongly disagree, 10 = strongly agree) [45].

##### Patient-Reported Outcomes

The EORTC Quality of Life-Core 30 (EORTC QLQ-C30) measured health-related quality of life using a 30-item core survey [46]. Fatigue was assessed using the Functional Assessment of Chronic Illness Therapy-fatigue subscale (13-items) [47]. The Hospital Anxiety and Depression Scale evaluated depression and anxiety (14-items), and the Pittsburgh Sleep Quality Index questionnaire measured sleep quality [48,49,50]

Socio-cognitive determinants of physical activity (intervention mechanisms):

Barrier self-efficacy (9 items) [51], outcome expectations (8 items) [52], motivation type (19 items) [53], social support (2 items) [51], intentions (4 items) [54], behavioural capability (3 items) [39] and habit formation (4 items) [31] were all assessed at baseline and follow-up.

##### Objective Measures of Physical Function and Muscular Strength

Face-to-face measures assessing aerobic fitness, muscular strength and ambulation were completed on a sub-group of participants to examine the feasibility of conducting face-to-face testing as part of the trial design and to understand preliminary efficacy of the *ExerciseGuide* intervention on physical function and muscular strength. Selection was based on proximity to the University of South Australia testing site (Adelaide). Aerobic fitness was measured with the time taken (seconds) to complete a 400-m walk (on a 20-m track) [8]. The timed up-and-go test (3 m track) and the repeated chair stand (5 repetitions) were measured by time to completion to provide physical functioning scores [55,56]. Tests were performed in triplicate (except 400 m) with recovery time between trials. Dynamic muscle strength was assessed with the one-repetition maximum method. The leg extension and chest press exercises were used to determine lower and upper limb strength, respectively. Patients with proximal femur bone lesions were excluded from the leg extension one-repetition maximum test. Those with rib, thoracic spine and humerus lesions were excluded from the chest press one-repetition maximum test [8].

#### 2.6.5. Trial Feasibility

Feasibility was evaluated using screening, recruitment and attrition data, as well as the proportion of participants with complete data for each outcome measure. As an approximate representation of intervention delivery expense, the time taken to deliver coaching sessions and respond to questions was collected.

### 2.7. Sample Size

The target sample size for this study was 66. This sample size was considered sufficient, based on previous similar pilot studies to reasonably estimate performance against our pre-specified criteria for success [57,58]. A more detailed overview of sample size determination is provided in the study protocol [21].

### 2.8. Data Analysis

Study data was analysed using SPSS version 26 (IBM, Chicago, IL, USA). Descriptive statistics were calculated for all outcome measures by study arm and were expressed as means and standard deviations and as medians and range if the distribution was skewed. Categorical data was presented as a frequency (percentage). Efficacy outcome measures were compared between groups using analysis of covariance (ANCOVA) where baseline values and treatment status were the covariates. In all analyses residual distributional assumptions were checked and if appeared violated, the data was natural log transformed. Randomised participants were analysed in the group they were allocated to. All analyses were performed with complete cases. Significance was set at 0.05 two-sided and no adjustments were made for multiple testing. Qualitative data from open-ended questions were analysed for common themes as well as feedback for intervention refinement.

## 3. Results

### 3.1. Participants

**Figure 2:**
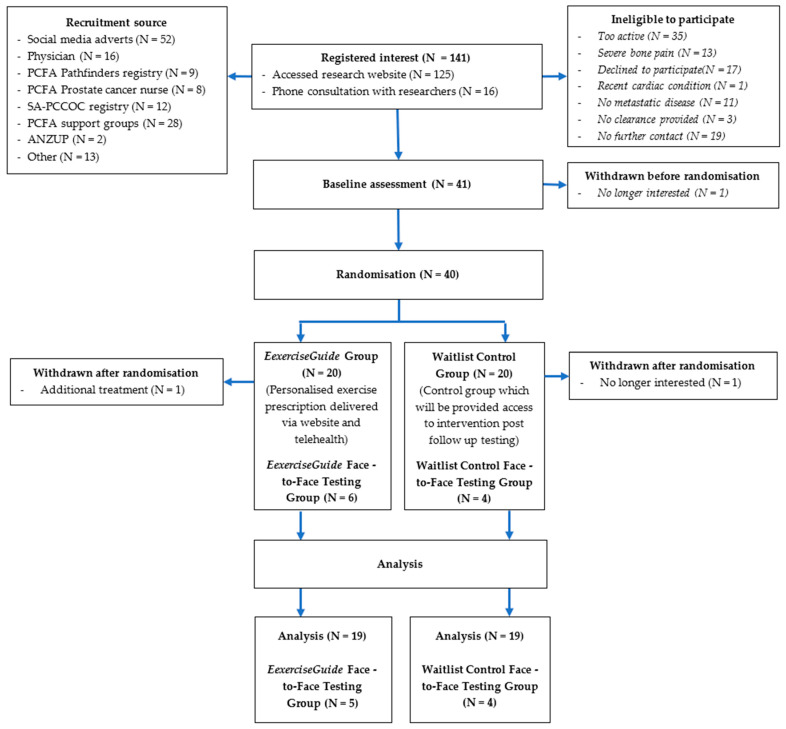
Participant flow chart.

**Figure 3:**
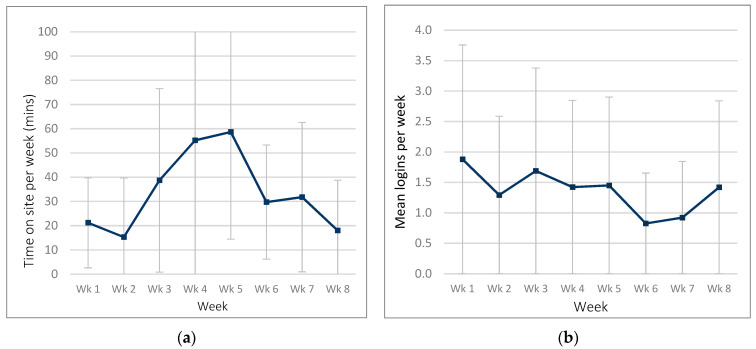
Website usage in the *ExerciseGuide* intervention group: (**a**) time spent on website per week and (**b**) number of logins per week.

Participant flow through the study is outlined in Figure 2. A total of 141 individuals screened themselves for eligibility on the *ExerciseGuide* website or were directly contacted and were screened by a researcher (HELE) over the 12-month period (scheduled date of closure). Of that, 41 eligible participants provided written informed consent, and medical clearance (with any metastasis locations listed) and 40 were then randomised to either the control group (*n* = 20) or intervention group (*n* = 20). One participant withdrew before finishing the baseline testing, and two participants withdrew during the intervention, as explained in Figure 3. The prespecified recruitment goal of 66 was not met. Participant characteristics are presented in Table 1. Participants had a mean age of 70 ± 8.5 years, all identified as male and were approximately 3 ± 3 years since metastatic prostate cancer diagnosis. Over 80% had greater than one bone metastasis, a majority (93%) were currently undergoing androgen deprivation therapy, and approximately half had undergone chemotherapy (56%) and radiotherapy (46%). At baseline, participants completed 241.5 ± 154.7 min of moderate-to-vigorous physical activity per week and 1.3 ± 1.9 sessions of resistance training per week (total of 12.4 ± 19.1 min per week and a rate of perceived exertion of 0.5 ± 0.9.

### 3.2. Intervention Acceptability

The median score of the CSQ-8 was 28.0 (range = 16–31) out of 32, indicating a high level of intervention satisfaction and was above the pre-defined cut-points for success (i.e., ≥20). The system usability scale mean score was 67.0 ± 15.1 out of 100, which is one point under the pre-defined cut-point of 68 out of 100 (see Figure S1 for more detail). The overall median score of the perceived environmental supportiveness scale (PESS) was 6.5 out of 7.0 (range = 4.3–7.0). The subscores of the PESS, autonomy, structure and involvement were rated as 6.2 (range = 4.3–7.0), 6.6 (range = 3.2–7.0) and 6.2 (range = 4.6–7.0), respectively, out of 7. Website perceived relevance was high, with a score of 6.0 out of 7.0 (range = 1.7–7.0). Lastly, the individual module star ratings ranged from 3 to 4.5 out of 5, which is above average except for tracking modules 2 and 3 (Table 2).

Eighteen participants from the *ExerciseGuide* group provided written feedback regarding the intervention in the follow-up questionnaire. The support provided by the intervention and exercise physiologist was seen as a highlight by half of the participants (*N* = 9) and the structure of the program was noted as a positive by six participants, with comments including “the program got me out of the chair with definite plan in mind” and “the program is very structured, there is lots of information on how and why to exercise”. Constructive feedback included lack of exercise prescription variety and boredom (*N* = 4), navigation and usability issues (*N* = 3) and inability to adhere to the program (*N* = 3). Finally, potential improvements suggested by participants included ability to autogenerate multiple programs for diversity or increase the range of exercises (*N* = 4), increased personalised support from the exercise physiologist (*N* = 3) by increasing “regular contact” to help increase both adherence and supervision. See Tables S1–S3 for more detailed feedback.

### 3.3. Usage: Behavioural Outcomes Relating to Engagement

#### 3.3.1. Website Usage

*ExerciseGuide* participants spent an average of 93.3 ± 101.6 min (range = 4.3–373.6) on the site over the eight-week period. Participants logged into the website 6.1 ± 5.9 times (range 1–22) over the intervention. Weekly logins were highest in week one and week three, as seen in Figure 3.

#### 3.3.2. Website Modules Completed

The percentage of participants who viewed each module is shown in Table 2. There was a 100% (*n* = 20) completion rate in the Getting Started and Exercise Plan 1 (week 1–3) modules. The remaining modules had moderate competition rates, ranging from 45% for the Extra Help module to 75% for the Exercise Benefits module. There was a decrease in completing tracking modules over time (65% started the tracking module, 10% progressed through to week three of tracking and 0% completed the tracking module). The library function was used by 50% of the *ExerciseGuide* group, with an average usage time of 5.3 ± 6.4 min. Only one participant used the “Ask an Expert” feature.

#### 3.3.3. Self-Reported Adherence to Exercise Prescription

The exercise diary was completed and returned by 89% (*N* = 17) of the intervention group. The diaries indicated that participants had a self-reported resistance training session attendance (sessions completed divided by sessions prescribed) 64.6 ± 40.2% and aerobic training session attendance of 102 ± 62.7%. The self-reported volume adherence in resistance training (total sets × total repetitions prescribed per session) was 78.3 ± 77.9% and 91.5 ± 56.5% in aerobic training (total time prescribed per session). The perceived exercise intensity was reported as 6.6 ± 0.9 out of 10 in the resistance exercise sessions and 6.6 ± 1.1 out of 10 in aerobic sessions. In terms of self-perceived adherence, participants rated their adherence to their aerobic exercise program as 6.1 ± 3.3 out of 10 and 5.4 ± 3.8 out of 10 for their resistance training at follow-up.

#### 3.3.4. Telehealth Consults and Time

In week one, the mean telehealth consult time per participant was 25.15 ± 7.80 min. Telehealth mode varied; 50% of participants requested a Zoom call (*N* = 10), 40% of participants favoured a phone call (*N* = 8) and 5% used Skype (*N* = 1). The mean consult time for the week four telehealth consult was 23.92 ± 7.47 min. One participant switched from zoom to a phone consultation, and one participant withdrew from the study. One participant chose not to engage in the consultations due to time limitations.

Over the intervention, the exercise physiologist sent 65 text messages and 81 emails to support participants. Furthermore, 11 phone calls were completed throughout the intervention when participants requested or if participants did not reply to more than two messages in a row. The mean call time per participant was 11.50 ± 8.46 min, and the total call time per participant was 92.38 ± 8.43 min.

### 3.4. Safety

On follow-up, 85% of participants reported no (grade zero) bone pain (*N* = 16), 10% reported mild (grade one) bone pain (*N* = 2), and 5% reported moderate (grade two) bone pain resulting from the intervention (*N* = 1) (see Table S4). There were no grade three/life-threatening or serious adverse events resulting from participating in the *ExerciseGuide* intervention and as such met the criteria for success. The self-reported exercise diaries showed a severity of bone pain post resistance exercise of 0.3 ± 0.8 out of 10, with a 0.0 ± 0.2 change from pre-exercise levels. The severity of bone pain post aerobic exercise was 0.4 ± 0.8 with a 0.1 ± 0.2 increase from pre-exercise levels. Participants also reported non-bone pain post resistance exercise of 0.7 ± 0.9 and non-bone pain post aerobic exercise of 0.6 ± 0.9.

### 3.5. Efficacy

#### 3.5.1. Physical Activity

Between-group differences for all physical activity-related outcomes are presented in Table 3. In brief, accelerometer assessed moderate-to-vigorous physical activity (MVPA) differed between groups at follow-up by 10.0 min per day (95% CI = (1.3, 18.6); *p* = 0.01) in the *ExerciseGuide* group compared to the control. An increase observed in the *ExerciseGuide* group, and a reduction observed in activity per day in the control group accounted for this difference. The adjusted mean difference per day is sufficient to meet our efficacy criteria. When assessing weekly physical activity, accelerometry data showed a group difference of 69.9 min in favour of the intervention group compared to the control (95% CI = (15.1, 124.8); *p* = 0.01). No changes were seen in light or vigorous physical activity or sedentary activity (Table 3). Step count also differed between groups (*p* = 0.02). Self-reported exercise values did not differ significantly between groups at follow-up; however, resistance training duration (mins) increased by 10.3 min between groups at follow-up in favour of the intervention group and this change was borderline not significant.

#### 3.5.2. Computer-Tailored Individualised Exercise Prescription

Based on the data provided by participants, the *ExerciseGuide* resistance exercise programming for weeks 1–3 resulted in a mean frequency of 2.0 ± 0.0 sessions per week, and 6.4 ± 1.7 (range = 4–8) exercises prescribed per person. Overall, an RPE of six out of ten was prescribed for 68% of participants and seven out of ten for 32% of participants. The average weekly resistance exercise volume (total sets × total repetitions) was 1001.7 ± 291.0 units. The exercises prescribed are shown in the Table S5. The individualised aerobic programming in weeks 1–3 prescribed an average of 2.5 ± 0.5 sessions per week and a mean duration of 28.3 ± 4.9 (range = 10–36) minutes per session. An RPE of six out of ten was prescribed for fourteen participants (74%) and seven out of ten for five (26%) participants. A summary of the aerobic modes and flexibility exercises prescribed are shown in Tables S6 and S7). 

At the end of week three, self-reported responses to questions allowed for total exercise volume modifications based on reported compliance. In weeks 4–8 (exercise plan 2 module), the resistance algorithm prescribed 6.3 ± 1.8 (range = 4–8) exercises per person, and the rate of perceived exertion prescription did not change. The average session frequency per week increased to 2.5 ± 0.3, and the average weekly resistance exercise volume (total sets × total reps) was 1358 ± 515 units. The exercise plan 2 module prescribed 2.7 ± 0.5 aerobic sessions for weeks 4–8, 27.7 ± 7.6 min per session and one participant’s rate of perceived exertion changed from six to seven out of ten.

### 3.6. Secondary Outcomes

#### 3.6.1. Patient-Reported Outcomes

When controlling for baseline and active treatment, there were no differences detected between groups at follow-up in self-reported quality of life (*p* = 0.22–0.86), see Table 4. Whilst not statistically significant, the reduction in fatigue levels (5.3; 95% CI = (−0.4, 11.1); *p* = 0.06) and depression levels (−1.3; 95% CI = (−2.4–−2.4); *p* = 0.06) reported in the intervention relative to the control may be clinically relevant [47,59]. Lastly, over time, there were no statistical differences in sleep quality and anxiety between the *ExerciseGuide* and control groups.

#### 3.6.2. Mechanisms of Action

Changes in social cognitive determinants of exercise between groups adjusted for baseline and active treatment are presented in Table 5. Barrier self-efficacy was lower among *ExerciseGuide* group participants compared to control group participants for both aerobic (−3.9; 95% CI = (−8.2, 0.3); *p* = 0.07) and resistance training (−3.50; 95% CI = (−10.7, 2.3); *p* = 0.08) at follow-up. This finding was borderline not significant. There were positive effects on motivation type with greater identified regulation (i.e., driven by exercise being seen as personally important) observed in the intervention group relative to the control at follow-up. However, the mean difference was relatively small (0.4; 95% CI = (0.0, 0.7); *p* = 0.04). There was also a trend showing greater intrinsic motivation (motivated by exercise being inherently satisfying) in the intervention group in comparison to the control group. This difference was also relatively small (0.3; 95% CI = (0.0, 0.7); *p* = 0.07).

### 3.7. Sub-Group (Physical Function and Muscular Strength Measures)

In total, 27.5% (*N* = 11) of the total participant group were eligible to complete the subgroup measures prior to group randomisation. Of that, 90.9% agreed to participate in had been randomised into the intervention group, and four were randomised into the control group. Despite the very small sample size, there was a significant difference in 400 m walk time favouring the intervention group when comparing baseline to follow-up (0.8 min; 95% CI = −1.5, −0.1; *p* = 0.02). A significant mean difference of 8.5 kg, 95% CI = 0.9, 16.0, *p* = 0.04 was also seen in the one-repetition maximum chest press. This difference is clinically meaningful [60]. No other significant differences were observed between groups over the 8-week intervention (see Table S8).

## 4. Discussion

This study examined the acceptability, safety and preliminary efficacy of *ExerciseGuide* to determine if progression to a larger scale trial is merited. There were four important findings: (1) three of the four (acceptability, safety and meaningful moderate-to-vigorous physical activity participation) pre-specified criteria for intervention success were achieved; (2) intervention usability was just below criteria despite a high level of online engagement; (3) findings for the mechanisms of action were mixed; (4) criteria for trial feasibility were met except for recruitment.

The high levels of intervention acceptability observed in this pilot study are encouraging. While our sample likely suffers from some self-selection bias, it is notable that our study population is inclusive of older adults and others often perceived to have difficulties accessing online programs (e.g., those living in remote Australia) [61]. It is also notable that positive results were observed across multiple facets of acceptability, including those associated with uptake and efficacy (e.g., relevance) [37,62]. Of exception, intervention usability was deemed marginal, scoring 67.0 ± 15.1 out of 100. The score was one point under the pre-specified cut point for success, suggesting that the *ExerciseGuide* website performed just at or below average compared to industry standards. Similar scores have been observed in evaluations of other web-based physical activity websites in similar populations. Alley et al. reported a usability score of 62.9 ± 10.2 in a study evaluating a tailored website with interactive features for older Australian adults [63]. Finlay et al. compared usability of a computer-tailored website among Australian men with localised prostate cancer set up to deliver modules either in a free choice scenario (similar to *ExerciseGuide*) or a tunnelled scenario where a new module was unlocked each week. Average usability was higher in the tunnelled intervention 67.4 ± 14.6 compared to the free choice intervention 56.4 ± 12.2 [63,64], but as in the current study the score was marginal. Usability testing and iterative refinement was undertaken with *ExerciseGuide* in a small lab-based study to enhance usability with some success [17]. However, given, that usability is likely to affect the uptake and continued use of web-based tools additional strategies should be considered, including implementation of feedback from pilot study participants [65]. Findings in the Finlay study and qualitative feedback from two *ExerciseGuide* participants consider that usability may improve if participants are navigated through future interventions [64]. This may also lead to increased usage of education modules which were not accessed as often as the exercise prescription modules in the current study. Lastly, further revision to the exercise prescription component of the intervention may improve the acceptability of the intervention. Real world usability testing with iterative changes may be useful to ensure the changes are positive.

Prior to the study, another unknown aspect was the safety of a distance-based exercise prescription tool in individuals with metastatic prostate cancer. The current study reported zero serious adverse events (≥3 CTCAE grading criteria) and twelve study-related grade one events (increased muscular or joint soreness and acute fatigue levels). A recent systematic review in individuals with bone metastases supports the use of unsupervised exercise as long as there is a supervised component, including face-to-face exercise instruction [66]. The novel use of distance-based supervision may provide another safe method of instruction by qualified practitioners.

In terms of preliminary efficacy, the *ExerciseGuide* intervention resulted in additional 10.0 min (95% CI = (1.3, 18.6); *p* = 0.01) of moderate-to-vigorous physical activity minutes per day compared to the wait-list control group, which equates to 70 min per week. Our results are moderately higher than other web-based studies that included individuals with metastatic prostate cancer, including Trinh et al., who showed a 44.1 min (11.1 to 77.0) improvement over 12 weeks and Golsteijn et al. reported a raw score change of 60 min within the OncoActive intervention [15,67]. The additional physical activity increase observed in the current study may be due to increased individualised support through telehealth consults or multi-modal exercise prescription tailored to individuals’ current level of physical activity and metastases/injury locations, which were not available in the Trihn et al. or Golsteijn et al. studies. Although it is important to note that the current study was only eight weeks in duration, it is unknown what effect increased intervention length may have on key factors such as moderate-to-vigorous physical activity, physical fitness, fatigue and depression. There is limited research on optimal duration to see significant changes in this population and further research is required.

A highlight of the current study is the measurement of changes in targeted theoretical constructs to improve understanding of why the *ExerciseGuide* intervention changed moderate-to-vigorous physical activity behaviours, which can have implications for future research. There was an increase in identified motivation in the *ExerciseGuide* group, which indicates a rise in individuals performing exercise because they are driven by reasons personally important, which may be indicative of using computer-tailoring algorithms to provide personally relevant messages [38]. A review by Teixeira et al. examining the association between motivation type (based on self-determination theory) and uptake and maintenance of exercise found that increased identified motivation is associated with exercise adoption [30]. As such, *ExerciseGuide* may be a promising intervention for engaging individuals with metastatic prostate cancer in exercise. However, additional strategies may be needed to encourage exercise maintenance as there were large variations in attending and completing prescribed exercise among participants over the 8-week intervention. The review by Teixeira et al. suggested that it is an increase in intrinsic motivation that is associated with longer term exercise participation. In our study, there was a positive trend, but no significant difference compared to the control in intrinsic motivation. Strategies to increase exercise enjoyment, such as affect regulated exercise [68] and a greater variety of exercises may be useful.

Previous research has also suggested that habit strength and self-efficacy are important for maintenance of exercise behaviours [29]. Our intervention did not improve habit strength relative to the control. There was a trend towards reduced self-efficacy in the *ExerciseGuide* group in comparison to the control group despite introducing behaviour change techniques to improve self-efficacy, including allowing participants to adapt their prescription based on perceived confidence levels, video demonstrations showing how to perform the behaviour, psycho-education around goal-setting, planning and obtaining further support, and by facilitating self-monitoring [26,69]. Interestingly, reductions in self-efficacy have been seen in other web-based physical activity interventions in individuals diagnosed with cancer. Forbes et al. suggest that if participants have not recently undertaken a physical activity regime, their personal beliefs regarding physical activity such as ease or acute benefits may be overestimated [26]. Once they undertake the program, their beliefs may become more realistic within their current constraints [26]. Given intrinsic motivation, habits scores and self-efficacy are predictive of long-term exercise adherence [30], changes to the *ExerciseGuide* intervention may be needed to support exercise maintenance. Evaluating behavioural determinants during the intervention and targeting strategies to improve these constructs over time is recommended.

The difference between the self-reported aerobic and resistance adherence (102 ± 62.7% vs. 64.6 ± 40.2%) and volume (91.5 ± 56.5% vs. 78.3 ± 77.9%) was interesting. A possible explanation for the disparity may be due to the higher levels of aerobic exercise behaviours at baseline. Participants may have found it easier to adhere to aerobic exercise in comparison to resistance training as it was a modality that they were already completing in some form. Therefore, participants in distance-based exercise interventions such as *ExerciseGuide* may require more targeted support to develop resistance training routines, such as increased real-time remotely supervised sessions or tools to track, prompt and reward resistance training behaviours. Some changes to the resistance training prescription library to increase variety may also improve adherence through increased enjoyment. Participant qualitative feedback suggested increased variety of resistance-training exercises would be appreciated for this reason. Another strategy that may work to increase adherence could be providing opportunities for participants to complete their individualised program in remotely supervised online group classes using a telehealth platform [70]. This could increase feelings of relatedness, which according to self-determination theory, would enhance intrinsic motivation [71]. The authors conclude there is reasonable feasibility of conducting a larger-scale trial. Pre-specified criteria were surpassed for the proportion of outcome data collected and participant retention. The study did not meet the pre-specified criteria of recruiting 66 participants; however, recruitment source was tracked, and lessons learned from the trial should increase recruitment feasibility for future studies. In terms of enrolled participants, recruitment from support groups (free), physicians (free) and paid social media advertisements (USD 81 per person recruited) were most cost-effective. In contrast, paid-registry recruitment was not as cost-effective (USD 800 per person recruited). We had few physicians/hospital sites involved and increasing this would likely lead to increased feasibility. Overall, evidence-based strategies to enhanced recruitment would be welcome. For example, Frampton et al. recommended that future researchers embed recruitment trials within randomised trials to help us better understand the efficacy of recruitment strategies in terms of increasing uptake and recruiting representative samples [72].

### Strengths and Limitations of the Study

The current pilot study should be interpreted within the context of key strengths and limitations. The recruitment goal of 66 participants was not met, which may have implications on future trial design [58]. The study was eight weeks in duration, and there was no long-term follow-up. As such, authors could not determine whether the intervention resulted in long term changes of key parameters such as moderate-to-vigorous activity levels. Despite a diagnosis of metastatic cancer, participants who were reasonably active prior to the intervention may not be representative of all individuals with metastatic prostate cancer. Finally, self-report measures were used for various study outcomes, such as intervention adherence, which may introduce response biases.

A strength of the study is that the *ExerciseGuide* intervention was methodically developed based on behavioural theory, previously investigated exercise prescription methodology, and formative user-centred research [5,8,17]. Furthermore, the study used a randomised design and publication of the study protocol aimed to ensure transparency around pre-specified criteria for success [21]. Lastly, the study examined different measures of acceptability and mechanisms of action, which has provided the researchers with the opportunity to explore what intervention components may be efficacious and improve future intervention development.

## 5. Conclusions

Our findings provide preliminary evidence that a web-based tailored exercise and behavioural change intervention designed for individuals with metastatic prostate cancer is acceptable, safe and efficacious for improving moderate-to-vigorous activity levels, fatigue and motivation. Given the high disease burden in this population, there is a clear need to develop effective distance-based and scalable supportive care interventions for individuals who are unable to access supervised interventions. As such, we consider that a large-scale trial with an enhanced version of the intervention is justified.

## Figures and Tables

**Table 1:** Participant characteristics for the whole sample at baseline (*N* = 40).

Characteristics		Intervention *N* = 20	Control *N* = 20	Total *N* = 40
Age, mean ± SD, year		69.5 ± 6.6	70.8 ± 10.2	70.2 ± 8.5
Weight, mean ± SD, kg		95.9 ± 20.8	90.0 ± 17.1	92.9 ± 19.0
Body Mass Index, mean ± SD, kg/m^2^		30.5 ± 5.2	28.7 ± 5.2	29.6 ± 5.3
Marital status, *N* (%)	Married/de facto	15 (75.5%)	13 (61.9%)	28 (68.3%)
	Widowed	1 (5.0%)	2 (9.5%)	3 (7.3%)
	Separated	4 (20.0%)	2 (9.5%)	6 (14.6%)
	Single	0 (0.0%)	4 (19.0%)	4 (9.8%)
Location, *N* (%)	Major city	12 (60.0%)	14 (66.7%)	26 (63.4%)
	Inner regional	5 (25.0%)	4 (19.0%)	9 (22.0%)
	Outer regional	1 (5.0%)	3 (14.3%)	4 (9.8%)
	Remote or very remote	2 (10.0%)	0 (0.0%)	2 (4.8%)
Education, *N* (%)	Secondary School	2 (10.0%)	8 (38.1%)	10 (24.4%)
	Trade/TAFE	11 (55.0%)	5 (23.8%)	16 (39.0%)
	University/Other Tertiary	7 (35.0%)	8 (38.1%)	15 (36.6%)
Employment, *N* (%)	Employed full-time	1 (5.0%)	2 (9.5%)	3 (7.3%)
	Employed part-time	1 (5.0%)	2 (9.5%)	3 (7.3%)
	Self-employed	0.0 (0%)	3 (14.3%)	3 (7.3%)
	Unemployed	2 (10.0%)	1 (4.8%)	3 (7.3%)
	Retired	16 (80.0%)	13 (61.9%)	29 (70.8%)
Current treatment, *N* (%)	Surgery	0 (0.0%)	0 (0.0%)	0 (0.0%)
	Radiotherapy	2 (10.0%)	2 (9.5%)	4 (9.8%)
	Chemotherapy	6 (30.0%)	7 (9.5%)	13 (31.7%)
	Hormone therapy	19 (90.0%)	19 (90.5%)	38 (92.7%)
Previous treatment, *N* (%)	Surgery	8 (40.0%)	5 (23.8%)	13 (31.7%)
	Radiotherapy	9 (45.0%)	10 (47.6%)	19 (46.3%)
	Chemotherapy	9 (45.0%)	14 (66.7%)	23 (56.1%)
	Hormone therapy	1 (5.0%)	1 (5.0%)	2 (9.5%)
Current PSA, mean ± SD,		15.2 ± 31.9	10.2 ± 38.7	12.5 ± 35.2
Time since metastatic disease diagnosis, mean ± SD, years		3.5 ± 3.1	2.57 ± 3.1	3.0 ± 3.1
Number of individuals with ≥1 bone lesion, *N* (%)		15 (75.0%)	18 (85.7%)	33 (80.5%)
Number of co-morbidities, mean ± SD		1.5 ± 1.6	2.0 ± 1.6	1.7 ± 1.6

Abbreviations: SD, standard deviations; *N*, number; PSA, prostate specific androgen.

**Table 2:** *ExerciseGuide* website usage within the intervention group (*N* = 20).

Module Name	Percentage of Modules Viewed		Average Total Time in Module per Participant (mins)		Average Total Page Views		Star Ratings		
	(%)	(*N*)	(M)	(SD)	(M)	(SD)	(Median)	(Range)	(*N*)
Introduction	100%	20	2.4	2.3	6.7	4.5	Nil	Nil	0
Exercise plan (Week 1–3)	100%	20	19.5	21.0	18.9	13.4	4.0	4.0–4.0	3
Exercise plan (Week 4–8)	65%	13	5.2	6.0	5.7	6.0	3.0	3.0–3.0	1
Exercise benefits	75%	15	2.0	1.0	3.7	2.5	4.0	4.0–5.0	4
Exercise safely	60%	12	4.0	3.0	1.5	1.5	4.5	4.0–5.0	6
Make it last	55%	11	1.5	1.3	2.0	2.2	4.0	4.0–5.0	3
Exercise+ (lifestyle)	50%	10	4.3	3.4	1.3	1.6	4.0	4.0–4.0	2
Extra help	45%	9	1.3	1.3	1.2	1.4	4.0	4.0–5.0	3
Weekly tracking module 1	65%	13	3.1	2.2	3.0	2.7	3.0	1.0–5.0	5
Weekly tracking module 2	25%	5	2.0	1.1	0.7	1.2	2.5	2.0–3.0	2
Weekly tracking module 3	10%	2	1.2	1.1	0.2	0.7	2.0	4.0–4.0	4

Racking modules were available weekly. Tracking modules 4–8 were completed by 0% (*N* = 0) of the *ExerciseGuide* intervention group.Abbreviations: *N*, number; M, mean; SD, standard deviations.

**Table 3:** Group differences in physical activity measures over the course of the intervention.

Outcome	Baseline		Follow-Up		Adjusted Mean Differences	*p*-Value
	EG (*N* = 20)	CON (*N* = 20)	EG (*N* = 19)	CON (*N* = 19)	M (95% CI)	
MVPA (min/day)	30.57 ± 22.0	38.4 ± 22.2	35.1 ± 23.6	32.0 ± 22.7	10.0 (1.3, 18.6)	0.01 *
Sedentary activity (min/day)	668.1 ± 171.0	708.7 ± 66.5	693.6 ± 117.1	731.0 ± 66.5	−33.46 (−95.0, 28.1)	0.63
Steps (steps/day)	4977 ± 3146	6169 ± 3001	5885 ± 3071	5556 ± 3141	1332 (159, 2505)	0.02 *
Light PA (min/week)	469.5 ± 206.9	561.6 ± 159.8	544.9 ±230.4	526.2 ± 199.3	96.7 (−5.4, 198.8)	0.10
Moderate PA (min/week)	203.0 ± 149.7	256.9 ± 150.0	232.7 ± 158.8	208.6 ± 142.3	69.9 (15.1, 124.8)	0.01 *
Vigorous PA (min/week)	11.0 ± 14.1	13.4 ± 13.3	12.0 ± 16.0	15.6 ± 21.1	1.9 (−12.4, 8.5)	0.88
GLTEQ (aerobic)	37.9 ± 37.7	40.6 ± 29.2	52.3 ± 38.8	36.7 ± 28.0	16.9 (−0.5, 33.8)	0.07
Resistance training frequency (sessions/week)	1.4 ± 2.0	1.2 ± 1.8	2.3 ± 3.6	1.8 ± 2.0	0.5 (−0.8, 1.6)	0.90
Resistance training duration (min)	10.8 ± 17.9	14.0 ± 20.3	22.4 ± 18.2	12.5 ± 17.3	10.3 (−1.2, 21.7)	0.08
Resistance training RPE	0.4 ± 0.8	0.6 ±0.1	6.2 ± 18.9	4.2 ± 3.0	2.1 (0–0.4, 3.9)	0.13

Abbreviations: EG, *ExerciseGuide* group; CON, Control group; PA, Physical activity; GLTEQ, Godin leisure time questionnaire; RPE, rate of perceived exertion. * Indicates significant values (*p* < 0.05).

**Table 4:** Group differences in patient-reported outcome measures over the course of the intervention.

Outcome	Baseline		Follow-Up		Mean Difference	*p*-Value
	EG (*N* = 20)	CON (*N* = 20)	EG (*N* = 19)	CON (*N* = 19)	M (95% CI)	
Quality of life (EORTC QLQ-C30)						
Global Health status ^1^	62.7 ± 22.3	71.9 ± 15.0	68.4 ± 22.0	64.5 ± 22.2	9.3 (−3.7–22.4)	0.24
Functional status ^2^						
Physical functioning	84.1 ± 16.2	90.9 ± 12.0	85.9 ± 17.4	87.0 ± 12.5	4.1 (−2.8–10.9)	0.44
Role functioning	84.2 ± 22.5	82.5 ± 18.0	82.5 ± 26.9	75.4 ± 28.5	5.2 (−10.6–21.0)	0.37
Emotional functioning	86.8 ± 10.9	86.4 ± 16.2	84.6 ± 13.4	84.2 ± 18.4	−0.2 (−8.1–7.6)	0.86
Cognitive functioning	77.2 ± 18.6	85.1 ± 13.5	77.2 ± 18.6	81.6 ± 12.5	0.3 (−9.3–9.8)	0.81
Social functioning	78.9 ± 22.1	74.6 ± 21.1	80.7 ± 21.7	70.2 ± 27.0	8.4 (−5.7–22.6)	0.63
Symptom Scales ^3^						
Fatigue	36.3 ± 20.2	31.6 ± 21.7	39.8 ± 19.4	38.0 ± 23.0	2.5 (−7.2–12.2)	0.56
Nausea/Vomiting	3.5 ± 11.9	1.8 ± 7.6	0.9 ± 3.8	3.5 ± 8.9	−3.4 (−7.6–0.9)	0.22
Insomnia	36.8 ± 27.0	24.6 ± 24.4	36.8 ± 6.7	29.8 ± 29.2	−1.3 (−13.9–26.2)	0.27
Pain	21.1 ± 24.7	14.9 ± 19.2	24.6 ± 25.1	21.9 ± 26.7	−0.5 (−15.5–14.5)	0.81
Dyspnoea	14.0 ± 16.9	15.8 ± 17.1	15.8 ± 20.4	19.3 ± 16.9	−2.6 (−14.4–9.2)	0.40
Appetite loss	10.5 ± 15.9	1.8 ± 7.6	5.3 ± 16.7	12.8 ± 27.7	−11.7 (−27.5–27.5)	0.18
Diarrhoea	3.5 ± 15.3	5.3 ± 13.0	5.2 ± 12.5	13.0 ± 20.3	−7.7 (−19.0–3.5)	0.22
Constipation	3.5 ± 10.5	8.8 ± 18.7	7.0 ± 23.8	3.5 ± 10.5	0.5 (−1.0–2.0)	0.49
Financial difficulties	15.8 ± 23.2	10.5 ± 19.4	7.1 ± 17.8	14.0 ± 27.9	−9.6 (−23.9–4.6)	0.26
Fatigue (FACIT-F) ^4^	37.7 9.6	41.4 ± 6.5	38.4 ± 15.0	37.9 ± 12.4	5.3 (−0.4–11.1)	0.06
Depression (HADS-D) ^5^	3.3 ± 3.1	2.9 ± 1.9	3.1 ± 2.8	4.1 ± 2.3	−1.3 (−2.4–−2.4)	0.06
Anxiety (HADS-A) ^5^	2.9 ± 3.2	4.4 ± 2.7	3.2 ± 3.4	4.7 ± 3.3	−0.2 (−1.7–1.2)	0.74
Sleep Index (PSQI) ^6^	7.2 ± 2.9	6.9 ± 3.3	11.5 ± 3.7	10.7 ± 3.1	0.6 (−1.4–2.6)	0.10

^1^ Global health status/quality of life score ranged from 0–100, with a higher score representing a higher quality of life. ^2^ Scores for the functional scales ranged from 0–100, with a higher score representing a high level of functioning. ^3^ Scores for the symptom item ranged from 0–100, and a higher score represents a higher level of symptomatology/problems. ^4^ FACIT-fatigue, all items were summed to create a single fatigue score ranging from 0 to 52, with higher scores representing better functioning or less fatigue. ^5^ Hospital anxiety and depression scale score provides two subscale scores (HADS-D) and (HADS-A). A score greater than seven denotes anxiety or depression. ^6^ The PSQI total score can range from 0 to 21. A global score of five or more indicates poor sleep quality, the higher the score, the worse the sleep quality. Abbreviations: EG, *ExerciseGuide* group; CON, Control group.

**Table 5:** Differences in mechanisms of action by group at baseline and follow-up (*N* = 40).

Outcome	Baseline		Follow-Up		Adjusted Change Mean Difference (95% CI)	*p*-Value
	EG (*N* = 20)	CON (*N* = 20)	EG (*N* = 19)	CON (*N* = 19)		
Self-efficacy ^1^						
Barrier (aerobic) sum	36.2 ± 8.6	36.7 ± 6.7	33.5 ± 8.7	36.3 ± 5.7	−3.9 (−8.2, 0.3)	0.07
Barrier (resistance) sum	35.6 ± 8.6	35.8 ± 6.7	33.1 ± 7.7	36.5 ± 5.2	−3.50 (−10.7, 2.3)	0.08
Outcome expectations ^2^						
Sum	31.9 ± 4.0	31.4 ± 4.3	32.3 ± 4.5	31.7 ± 3.5	0.23 (−1.5, 2.0)	0.79
Motivation type ^3^						
Amotivation	0.6 ± 0.7	0.4 ± 0.8	0.3 ± 0.7	0.4 ± 0.5	−0.1 (−0.5, 0.2)	0.40
External regulation	0.9 ± 0.9	0.9 ± 1.0	0.6 ± 0.6	0.8 ± 1.0	−0.1 (−0.6, 0.3)	0.54
Introjected regulation	1.6 ± 1.2	1.9 ± 1.2	1.5 ± 1.0	1.9 ± 1.3	−0.1 (−0.6, 0.5	0.59
Identified regulation	2.6 ± 0.7	3.1 ± 0.7	2.8 ± 0.7	2.9 ± 0.9	0.4 (0.0, 0.7)	0.04 *
Intrinsic regulation	2.0 ± 1.1	2.7 ± 0.8	2.3 ± 0.9	2.5 ± 0.8	0.3 (0.0, 0.7)	0.07
Social support ^4^						
Sum	7.7 ± 2.3	6.7 ± 2.6	6.9 ± 2.2	7.4 ± 2.1	−1.0 (−2.5, 0.3)	0.14
Intention ^5^						
Aerobic intention strength	68.2 ± 27.1	67.7 ± 22.3	62.4 ± 27.7	68.4 ± 19.3	−7.2 (−20.8, 6.4)	0.29
Resistance intention strength	64.8 ± 31.9	66.6 ± 37.7	54.8 ± 30.6	67.3 ± 25.9	−14.7 (−30.5, 1.1)	0.06
Behavioural capability						
Aerobic training experience ^6^	2.5 ± 1.3	3.4 ± 0.8	3.0 ± 1.0	3.2 ± 0.9	0.2 (−0.4, 0.8)	0.47
Resistance training experience ^6^	1.7 ± 1.4	2.1 ± 1.3	1.7 ± 1.3	1.8 ± 1.4	0.2 (−0.3, 0.9)	0.37
Falls confidence ^7^	3.0 ± 0.7	3.0 ± 1.1	2.9 ± 1.2	2.9 ± 1.1	0.0 (−1.1, 0.4)	0.39
Habit formation ^8^						
Sum	12.9 ± 6.3	16.8 ± 6.1	13.8 ± 6.7	17.1 ± 6.1	−1.2 (−4.9, 2.3)	0.47

^1^ Barrier self-efficacy consisted of nine items which were scored from one (not very confident at all) to five (very confident) and the barrier self-efficacy sum is the summation of all nine item scores (ranging from 5–45). ^2^ Outcome expectation consisted of eight items which were scored from one (strongly disagree) to five (strongly agree) and outcome expectation sum is the summation of all eight item scores (ranging from 5–40). ^3^ Motivation type was determined by the Behavioural Regulations in Exercise Questionnaire (BREQ). Items were scored from one (not true for me) to five (very true for me). The mean score was calculated for every sub-item and scores range from 0–5. ^4^ Social support was the summation of two items (ranging from 2–14), which were scored from one (strongly disagree) to seven (strongly agree). ^5^ Intention was determined on a scale of 0 (no intention to exercise) to 100 (full intention to exercise). ^6^ Exercise experience for both aerobic and resistance training was scored on a scale of one (not true for me) to five (very true for me). ^7^ Confidence of not falling within the next 12 months was scored on a scale of one (not true for me) to five (very true for me). ^8^ Habit formation consisted of four items which were from one (strongly disagree) to five (strongly agree) and the habit formation sum is the summation of all four item scores (ranging from 5–20). * Indicates significant values (*p* < 0.05).

## Data Availability

The data presented in this study are available on request from Camille Short at camille.short@unimelb.edu.au.

## Supplementary Materials

The following are available online at https://www.mdpi.com/article/10.3390/cancers13235925/s1, Figure S1: System usability scores in the *ExerciseGuide* intervention, Table S1: Written positive qualitative feedback from *ExerciseGuide* intervention, Table S2: Written constructive qualitative feedback from *ExerciseGuide* intervention, Table S3: Written thoughts for improvement feedback from *ExerciseGuide* intervention, Table S4: Nonserious adverse events self-reported during *ExerciseGuide* intervention, Table S5: Resistance exercise prescribed within the *ExerciseGuide* intervention, Table S6: Aerobic exercise prescribed and completed within the *ExerciseGuide* intervention, Table S7: Flexibility exercise prescribed within the *ExerciseGuide* intervention, Table S8: Subgroup physical functioning measures at baseline and follow-up (*N* = 10).
