# Supplementary material for: Acceptability and Preliminary Efficacy of a Web- and Telephone-Based Personalised Exercise Intervention for Individuals with Metastatic Prostate Cancer: The ExerciseGuide Pilot Randomised Controlled Trial
Source: Cancers (Basel). 2021 Nov 25;13(23):5925. doi: 10.3390/cancers13235925 (PMC8656540; doi:10.3390/cancers13235925)
Supplement: Supplementary file 1 [file cancers-13-05925-s001.zip › cancers-1463862-supplementary.pdf]

cancers

MDPI

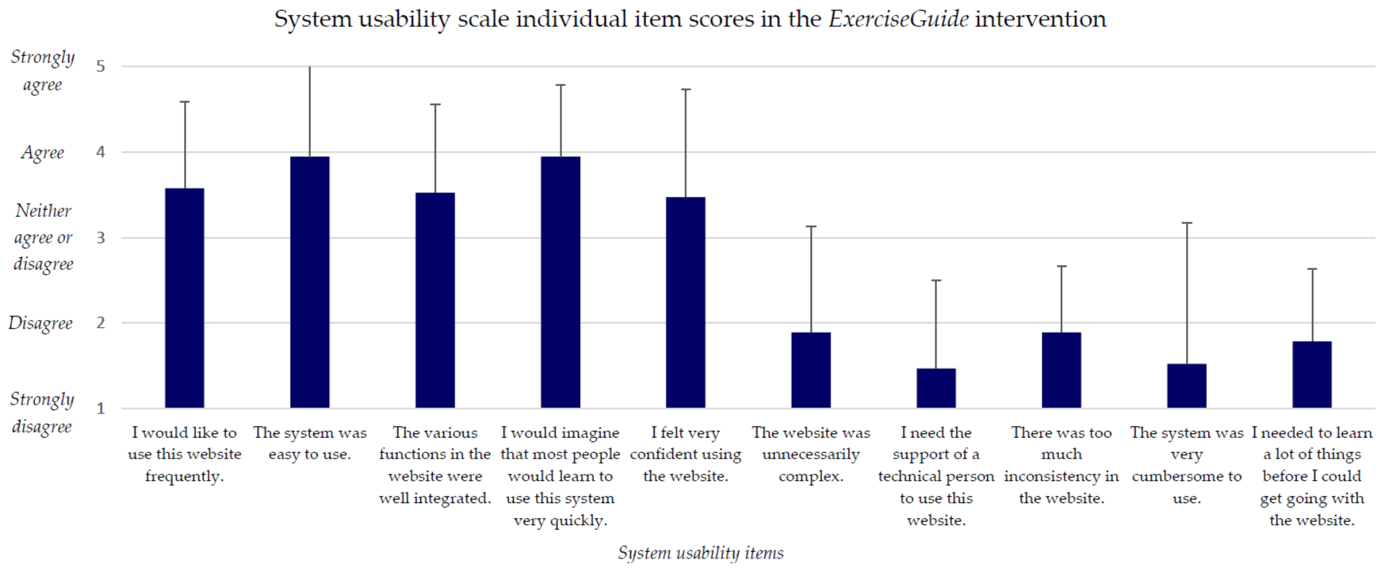

Figure S1. System usability scores in the *ExerciseGuide* intervention.

Table S1. Written positive qualitative feedback from *ExerciseGuide* intervention.

Theme	Participant quotes
Support	Having the AEP available allowed me to adjust the program as I progressed (EG01).<br>Someone available for support (EG11).<br>Regular contact from the exercise team (EG15).<br>The online face-to-face consultation was very good, plus the flexibility to adjust the program (EG30).<br>The interactive contact by the exercise physiologist (EG33).<br>Having a qualified physiologist who is enthused about the programme is a great asset (EG39).<br>Getting to do some exercise instruction at home with backup (EG40).<br>Provided (guilt) motivation. Helped me transition from inactive (EG47).<br>Helpful from a support viewpoint, encouraging to still be active (EG53).
Focus/structure	Made me focus on helping myself to achieve better wellbeing both physical and mentally (EG11).<br>The program is very structured, there is lots of information on how and why to exercise (EG20).<br>Program give you something to look forward to and you can set goals (EG37).<br>Got me out of the chair with definite plan in mind (EG50).<br>The program was very relevant and easy to follow (EG55).<br>Very precise and straightforward (EG11).
Instruction	The exercises and stretching modules clearly explained with a video to negate any doubts (EG39).<br>Good structure, clear instructions on how to complete the exercises (EG48).
Equipment	Easy to use and versatile bands (EG10).
Tailoring	Having a tailored program that allowed me to vary my effort when I needed to (EG01).
Tracking	Booklet to keep track of exercises (EG15).
Other	All exercise is good. I suppose (EG13).<br>I really can't comment as my participation has been nothing short of dismal (EG29).<br>A fantastic program and very beneficial. I lost weight and felt a lot better both mentally and physically and mentally and this was during the first outbreak of Covid19 and lockdown and a very traumatic time due to major family issues (EG01).<br>I cannot say enough about this program, brilliant work putting it together (EG01).

Table S2. Written constructive qualitative feedback from *ExerciseGuide* intervention.

Theme	Participant quotes
Lack of variety in exercise prescription	Exercise program lacks motivation. Is the same old set of exercises that have been known to be difficult to maintain for decades (EG13).<br>Only one program of exercises (EG10).

	The resistance program became boring (EG30).<br>Not enough variety (EG40).
Navigation and usability issues	The website was difficult to navigate unless you have IT experience (EG15).<br>It wasn't always clear when to do which parts. So, for example I thought it was sequential so didn't start the later modules until I had virtually finished the 8 weeks. A 'roadmap' to navigate the program might help (EG20).<br>I got mixed up between no of repetitions and number of sessions per week early on but that's probably because I 'didn't read the instructions' properly (EG50).
Information about why certain exercises prescribed.	Could not feel any benefit in one of the exercises (Leg Fallout) and could not find any reason why this was so on the web site (EG39).<br>Not always clear why exercises were suggested (EG48).
Adherence issues	I started the program during chemotherapy after having suddenly retired. The changing circumstances of my life complicated the way in which I was able to engage with the program. Too many things changing too quickly. I struggled to remain consistent (EG47).<br>In my case I had bad hip pain, which made it hard to complete the program (EG37).<br>Could not get enthusiastic about regimented artificial exercise. Considering that I am physically capable of doing everything I need to do, I have not been able to find the necessary enthusiasm for this program. This may not always be the case though and I would welcome the chance to become re-involved if and when I require help in the future (EG41).
Needed more support	No threat of punishment for not doing exercises (EG10).
Time consuming	Were too time consuming with all the breaks I needed to take (EG13).

Table S3. Written throughts for improvement feedback from *ExerciseGuide* intervention.

Theme	Participant quotes
Increased exercise variety	Auto generate multiple programs for diversity (EG10).<br>Increase the range of exercises i.e.: above head extension and other muscle groups, these could be in a section for an ongoing programme (EG39).<br>May be vary the exercise program (EG30).<br>More back/core strengthening exercises (so it's easier to get up off the floor) (EG50).
Increased support from exercise physiologist	I feel I would have benefited from more supervision/oversight (EG47).<br>Possibly a more regular contact with an exercise physiologist would enable slackers like me to adhere to the schedule a bit (EG30).<br>Provide more follow up telephone calls (EG53).
Ability to manipulate exercise program	I had to change my exercises due to some physical limitations (after week 4). However, the new exercises could not be incorporated in the online version of the program (EG30).<br>Clearer explanation of outcomes would be useful together with explanations of alternatives (EG48).<br>A trial initially to see which work for me, and how many repeats before setting a target program (EG13).<br>The ability to alter the exercise to suit you (EG40).
Usability concerns	A web or phone app to track your exercise progress (EG10).
IT literacy	I had some minor technical glitches - when printing all the pages of the exercise program in weeks 4-8, the detailed exercise plan is truncated on the right-hand edge (EG20).<br>Software needs an update, there were inconsistencies between my responses and what the program (EG39).
Increased socialisation	Maybe provide an opportunity for participants to meet in a social environment (EG55).
Improved usability	The rubber bands were awkward to use (EG13).<br>Need a video to follow with music (EG13).

Table S4. Nonserious adverse events self-reported during *ExerciseGuide* intervention.

Nonserious adverse events	Grade 1[1]	Grade 2[2]	Grade 3[3]	Grade 4[4]	Grade 5[5]	Total
Total adverse events, n	26	6	0	0	0	32
Adverse events related to preexisting conditions, n	14	6	0	0	0	20
Study-related adverse events, n	12	0	0	0	0	12

Specific Adverse Events, n		Grade 1		Grade 2		Grade 3		Grade 4		Grade 5		Total	
		AT	*RT*	*AT*	*RT*	*AT*	*RT*	*AT*	*RT*	*AT*	*RT*	*AT*	*RT*
Joint or bone pain	Pre-existing	1	2	1	1	0	0	0	0	0	0	2	3
	Study related	2	1	0	0	0	0	0	0	0	0	2	1
Muscle pain or injury	Pre-existing	3	2	1	1	0	0	0	0	0	0	4	3
	Study related	7	4	0	0	0	0	0	0	0	0	7	4
Fatigue	Pre-existing	2	2	1	1	0	0	0	0	0	0	3	3
	Study related	0	0	0	0	0	0	0	0	0	0	0	0

[1] Grade 1 Mild; asymptomatic or mild symptoms; clinical or diagnostic observations only; intervention not indicated. [2] Grade 2 Moderate; minimal, local or noninvasive intervention indicated; limiting age-appropriate instrumental ADL*. [3] Grade 3 Severe or medically significant but not immediately life-threatening; hospitalization or prolongation of hospitalization indicated. [4] Grade 4 Life-threatening consequences; urgent intervention indicated. [5] Grade 5 Death related to AE. Abbreviations: n = number; AT = aerobic training; RT = resistance training.

Table S5. Resistance exercise prescribed within the *ExerciseGuide* intervention.

Mode	Region	Exercise	Exercise Plan 1 Total number of times prescribed N (%)	Exercise Plan 2 Total number of times prescribed N (%)
Prescribed by *ExerciseGuide* website	Upper Limb	Seated Chest Press	3 (15%)	1 (5%)
		Seated Bicep Curl	6 (30%)	5 (25%)
		Seated Row	7 (35%)	5 (25%)
		Seated Shoulder Press	0 (0%)	0 (0%)
		Seated Shoulder Raise	0 (0%)	0 (0%)
		Seated Triceps Extension	10 (50%)	8 (40%)
		Standing bicep curl	7 (35%)	6 (30%)
		Standing Row	2 (10%)	0 (0%)
		Standing Shoulder Press	0 (0%)	0 (0%)
		Standing Shoulder Raise	0 (0%)	0 (0%)
		Incline Push Up	8 (40%)	4 (20%)
	Trunk	Seated March	10 (50%)	8 (40%)
		Leg Fallout	4 (20%)	3 (15%)
		Single Leg Lift	3 (15%)	2 (10%)
		Single Leg Lift with Extension	4 (20%)	3 (15%)
		Double Leg Lift	2 (10%)	0 (0%)
		Double Leg Hip Lift	5 (25%)	2 (10%)
		All Fours	0 (0%)	1 (5%)
		All Fours Progression	0 (0%)	0 (0%)
	Lower Limb	Seated Knee Extension	7 (35%)	5 (25%)
		Seated Hamstring Curl	11 (55%)	8 (40%)
		Sit to stand	0 (0%)	0 (0%)
		Partial Squat	6 (30%)	4 (20%)
		Squat	2 (10%)	0 (0%)
		Standing Calf Raise	17 (85%)	12 (60%)
Added in during telehealth consult	Upper limb	Seated row	1 (5%)	1 (5%)
	Lower Limb	Wall sit	0 (0%)	2 (10%)
		Isometric leg extension	0 (0%)	1 (5%)
		Seated knee extension	1 (5%)	1 (5%)
		Seated hamstring curl	1 (5%)	1 (5%)

Table S6. Aerobic exercise prescribed and completed within the *ExerciseGuide* intervention.

Mode	Exercise Plan 1 Week 1-3 (N=20)	Exercise Plan 2 Week 4-8 (N=19)[1]
Any option (no bone metastases)	7 (35%)	3 (16%)

Primary mode prescribed by website	Water walking	7 (35%)	6 (32%)
	Stationary cycling	4 (20%)	2 (11%)
	Walking	2 (10%)	2 (11%)
	No prescription	0 (0%)	6 (32%)
Secondary mode/s prescribed by website	Water walking	3 (15%)	1 (5%)
	Stationary cycling	9 (45%)	8 (42%)
	Walking	8 (40%)	6 (32%)
	Elliptical/climber	2 (10%)	2 (11%)
Actual aerobic prescription completed	Walking	12 (60%)	10 (53%)
	Stationary cycling	4 (20%)	4 (21%)
	Pool Walking	2 (10%)	1 (5%)
	Water aerobics	1 (5%)	1 (5%)
	Jogging	1 (5%)	1 (5%)
	Cross-trainer	1 (5%)	1 (5%)
	No prescription completed	4 (20%)	5 (26%)

[1] One participant withdrew prior to week 4 and was not prescribed an updated program (exercise plan 2).

Table S7. Flexibility exercise prescribed within the *ExerciseGuide* intervention.

		**Exercise Plan 1 Week 1-3 (N=20)**	**Exercise Plan 2 Week 4-8 (N=19)[1]**
Stretching program	Stretching program requested	19 (95%)	7 (37%)
Number of stretches prescribed	Mean ± SD	4.8 ± 2.3	4.3 ± 2.8
Stretches prescribed	Triceps	17 (85%)	6 (32%)
	Chest	17 (85%)	6 (32%)
	Quads	11 (55%)	6 (32%)
	Glutes	7 (35%)	2 (11%)
	Groin	11 (55%)	6 (32%)
	Hamstring	11 (55%)	6 (32%)
	Calf	18 (90%)	7 (37%)

Table S8. Subgroup physical functioning measures at baseline and follow up (N=10).

**Outcome**	**Baseline**		**Follow-up**		**Adjusted Change**	**P-Value**
	**EG**	**CON**	**EG**	**CON**	**Mean difference (95% CI)**	
400m walk (min)	5.49 ± 1.52 (N=5)	4.53 ± 1.36 (N=4)	5.23 ± 2.02 (N=5)	4.80 ± 1.94 (N=4)	-0.8 (-1.5 – -0.1)	0.02*
Timed up and go (s)	9.72 ± 3.04 (N=6)	7.10 ± 3.83 (N=4)	8.64 ± 3.08 (N=6)	7.29 ± 3.84 (N=4)	-1.0 (-2.5 – 0.5)	0.15
Repeated chair stand (s)	16.93 ± 7.05 (N=6)	13.30 ± 4.58 (N=4)	16.07 ± 6.00 (N=6)	13.22 ± 4.76 (N=5)	-0.7 (-2.1 – 0.6)	0.22
1RM – Chest press (kg)	58.75 ± 14.36 (N=5)	56.67 ± 11.55 (N=3)	65.00 ± 16.83 (N=5)	56.67 ± 11.55 (N=3)	8.5 (0.9 – 16.0)	0.04*
1RM – Leg extension (kg)	57.5 ± 25.0 (N=5)	50.0 ± 10.0 (N=4)	63.13 ± 21.35 (N=5)	53.3 ± 20.21 (N=4)	2.5 (-25.3 – 30.3)	0.82
